# Placental growth factor in assessment of women with suspected pre-eclampsia to reduce maternal morbidity: a stepped wedge cluster randomised control trial (PARROT Ireland)

**DOI:** 10.1136/bmj.n1857

**Published:** 2021-08-13

**Authors:** D Hayes-Ryan, A S Khashan, K Hemming, C Easter, D Devane, D J Murphy, A Hunter, A Cotter, F M McAuliffe, J J Morrison, F M Breathnach, E Dempsey, L C Kenny, K O’Donoghue, PARROT Ireland trial group, S Kappala, N Murphy, E Tully, M Dowling, J O’Leary, R Keane, A Murphy, B McElroy, E Snapes, D Hayes-Ryan, A S Khashan, K Hemming, M Daly, E Ní Bhraonáin, D Devane, D Murphy, A Hunter, A Cotter, F M McAuliffe, J J Morrison, F M Breathnach, E Dempsey, L C Kenny, K O’Donoghue, N Barry, N Buckley, K Corry, C Dunney, J Courtney, P Gargan, L Heaphy, A Kearney, L Murphy, M Murphy, C Mulcahy, C Ni Laighin, C Nolan, A Warde

**Affiliations:** 1 Irish Centre for Maternal and Child Health Research (INFANT), University College Cork, Cork, Ireland; 2 Cork University Maternity Hospital, Cork, Ireland; 3 School of Public Health, University College Cork, Cork, Ireland; 4 University of Birmingham, United Kingdom; 5 HRB Trials Methodology Research Network; 6 National University of Ireland, Galway, Ireland; 7 Trinity College Dublin & Coombe Women & Infants University Hospital Dublin 8, Republic of Ireland; 8 Royal Jubilee Maternity Hospital, Belfast, Northern Ireland; 9 University Maternity Hospital Limerick & University of Limerick; 10 UCD Perinatal Research Centre, School of Medicine, University College Dublin, National Maternity Hospital, Dublin, Ireland; 11 Department of Obstetrics & Gynaecology, National University of Ireland Galway; 12 Royal College of Surgeons in Ireland, Rotunda Hospital, Parnell Square W, Dublin 1, Ireland; 13 Department of Women’s and Children’s Health, Liverpool Women’s Hospital, University of Liverpool, UK; 14 Health Research Board Mother & Baby Clinical Trials Network Ireland; 15 Research & Innovation Department, University College Cork, Cork, Ireland; 16 Economics Department, University College Cork, Cork, Ireland

## Abstract

**Objective:**

To determine whether the addition of placental growth factor (PlGF) measurement to current clinical assessment of women with suspected pre-eclampsia before 37 weeks' gestation would reduce maternal morbidity without increasing neonatal morbidity.

**Design:**

Stepped wedge cluster randomised control trial from 29 June 2017 to 26 April 2019.

**Setting:**

National multisite trial in seven maternity hospitals throughout the island of Ireland

**Participants:**

Women with a singleton pregnancy between 20^+0^ to 36^+6^ weeks’ gestation, with signs or symptoms suggestive of evolving pre-eclampsia. Of the 5718 women screened, 2583 were eligible and 2313 elected to participate.

**Intervention:**

Participants were assigned randomly to either usual care or to usual care plus the addition of point-of-care PlGF testing based on the randomisation status of their maternity hospital at the time point of enrolment.

**Main outcomes measures:**

Co-primary outcomes of composite maternal morbidity and composite neonatal morbidity. Analysis was on an individual participant level using mixed-effects Poisson regression adjusted for time effects (with robust standard errors) by intention-to-treat.

**Results:**

Of the 4000 anticipated recruitment target, 2313 eligible participants (57%) were enrolled, of whom 2219 (96%) were included in the primary analysis. Of these, 1202 (54%) participants were assigned to the usual care group, and 1017 (46%) were assigned the intervention of additional point-of-care PlGF testing. The results demonstrate that the integration of point-of-care PlGF testing resulted in no evidence of a difference in maternal morbidity—457/1202 (38%) of women in the control group versus 330/1017 (32%) of women in the intervention group (adjusted risk ratio (RR) 1.01 (95% CI 0.76 to 1.36), P=0.92)—or in neonatal morbidity—527/1202 (43%) of neonates in the control group versus 484/1017 (47%) in the intervention group (adjusted RR 1.03 (0.89 to 1.21), P=0.67).

**Conclusions:**

This was a pragmatic evaluation of an interventional diagnostic test, conducted nationally across multiple sites. These results do not support the incorporation of PlGF testing into routine clinical investigations for women presenting with suspected preterm pre-eclampsia, but nor do they exclude its potential benefit.

**Trial registration:**

ClinicalTrials.gov NCT02881073.

## Introduction

Pre-eclampsia is a clinical manifestation of placental dysfunction. Complicating 2-8% of pregnancies, it is associated with significant maternal and neonatal morbidity and mortality.^1^ ^2^ The current diagnosis of pre-eclampsia is reliant on objective signs of end-stage disease such as maternal hypertension, significant proteinuria, abnormal biochemical or haematological indices, and ultrasound evidence of fetal growth restriction.^3^ ^4^ A robust diagnostic test for pre-eclampsia, and hence placental dysfunction, would prevent unnecessary hospitalisations and investigations for many pregnant women while also enabling earlier identification and focusing of resources on those who require it the most. Herein lies the potential of placental growth factor (PlGF) as a diagnostic biomarker for pre-eclampsia or placental dysfunction.^5^

As a vascular endothelial growth factor, PlGF regulates angiogenic events in pathological conditions.^6^ Circulating levels of PlGF in the maternal plasma increase in parallel with placental development, peaking at about 32 weeks’ gestation and then declining until birth.^7^ In pre-eclampsia, this rise and fall is considerably lower throughout pregnancy, and maternal plasma levels are significantly lower when the condition presents clinically. Observational studies have demonstrated the potential of PlGF in aiding diagnosis of pre-eclampsia in those presenting preterm with signs or symptoms of the disease.^8^ ^9^ ^10^ ^11^ However, an abnormal PlGF result may also prompt earlier intervention by clinicians, resulting in maternal benefit at the expense of the newborn, highlighting the need for adequately powered randomised controlled trials to determine the clinical utility and overall cost effectiveness of PlGF based testing.^12^

In 2016 the UK National Institute for Health and Clinical Excellence (NICE) published guidance on PlGF testing, in addition to clinical assessment, in women presenting with suspected pre-eclampsia from 20 to 34^+6^ weeks’ gestation. NICE advocated that PlGF testing should not be used to diagnose pre-eclampsia until further research was available on how an abnormal PlGF result would affect management decisions relating to timing of delivery and specifically to the perinatal outcomes resulting from decision making that may be influenced by PlGF testing.^13^ On the basis of this guidance, PlGF testing was not introduced into routine clinical care in Ireland.

Simultaneously to the UK PARROT trial,^14^ we set out to determine the efficiacy of PlGF in Ireland. The objective of this randomised trial was to evaluate the impact of knowledge of PlGF results on both maternal and neonatal outcomes. We hypothesised that the addition of PlGF measurement to current clinical assessment of women with suspected pre-eclampsia before 37 weeks' gestation would reduce associated maternal morbidity, without increasing neonatal morbidity, through improved risk stratification, earlier diagnosis, and targeted management of women with the disease.

## Methods

PARROT Ireland was an investigator-led, multicentre, stepped wedge cluster-controlled trial of PlGF measurement. The seven largest maternity units in Ireland were involved in this trial: the Coombe Women and Infants University Hospital Dublin, Cork University Maternity Hospital, University Maternity Hospital Limerick, the Royal Jubilee Maternity Hospital Belfast, University College Hospital Galway, the National Maternity Hospital Dublin, and the Rotunda Maternity Hospital Dublin. A cluster design, rather than individual randomisation, was chosen to facilitate a change in management at a hospital level rather than at an individual patient level, thus allowing the clinical influence of the additional test to be evaluated pragmatically.^15^ A stepped wedge design was chosen to increase the social acceptability of the trial to the hospitals and because a trial with just seven clusters risks baseline imbalance in a parallel design. Each of the maternity hospitals acted as a cluster. All clusters commenced the trial as a control, and in turn, each cluster transitioned at random to use the intervention at pre-specified time points. There was a short transition period of one week whenever a new cluster transitioned from control to intervention. During the transition period, a dedicated clinical research fellow remained at the site, to ensure the rollout of PlGF testing was conducted as per protocol and to familiarise staff locally with the new test. Once a cluster transitioned to the intervention, it remained using the intervention until the trial ceased. The trial was conducted following ethical principles that have their origin in the Declaration of Helsinki and are consistent with Good Clinical Practice and applicable regulatory requirements. Each local hospital ethics committee granted ethical approval. The trial was registered prospectively with Clinical Trials.gov, NCT02881073. A detailed description of the trial protocol and methodology has previously been published.^16^

### Population

Women presenting with suspected pre-eclampsia and a singleton pregnancy from 20 weeks and before 37 weeks' gestation were eligible for inclusion. Those with multiple pregnancies at any time point or those already diagnosed with pre-eclampsia were ineligible. Detailed inclusion and exclusion criteria are presented (supplementary tables S1 and S2). Eligible women were identified locally by researchers, approached individually, and written informed consent for inclusion in the study was obtained. Researchers were aware of the hospital's current randomisation when approaching eligible women, but the women themselves were blinded as to the hospital’s randomisation until after recruitment.

The trial statisticians developed a randomisation sequence for site transition with the order of site transitioning concealed from sites and principal investigators until 12 weeks before the site’s transition date. An allocation sequence was randomly selected (that is, a crossover order for the seven clusters) from a set of random sequences constrained so that the sum of the total cluster sizes in the intervention status was similar to the total sum of the cluster sizes in the control status. This restricted method of randomisation was used to provide a balance in total (expected) number of observations across intervention and control periods.^17^ ^18^ ^19^

As the intervention involved a blood test, blinding was not feasible, and both participants and investigators were aware of the participants’ assignment once enrolled in the study.

### Randomisation

Participants whose maternity hospital was randomised to the control arm received usual hospital care as per national guidelines (Health Service Executive or Institute of Obstetrics and Gynaecology Irish guidelines for those in the Republic of Ireland or the NICE guidelines for those in Northern Ireland).^3^ ^20^ As it was anticipated that most eligible participants would be recruited based on elevated blood pressure at presentation, the suggested management algorithm for the control arm of the trial was purposely based on the HSE and NICE guidelines for hypertension in pregnancy, which are based on the same research evidence and are similar in terms of key definitions and management recommendations (advocating outpatient care and withholding antihypertensives until blood pressure meets or exceeds 150/100 mm Hg and escalating frequency of review dependent of the level of hypertension present). 

In contrast, participants whose maternity hospital was randomised to the intervention had immediate maternal plasma PlGF quantified in addition to routine hospital investigations. The PlGF test was performed by an appropriately trained researcher at each site using a CE marked validated point of care platform, the automated Triage Meterpro (Quidel, San Diego CA, USA). The PlGF result was made immediately available to the participant and her clinical team as well as being documented clearly in the participant’s medical notes. A suggested further management algorithm was provided to the treating clinical team based on both the degree of hypertension present and the specific PlGF result (supplementary fig S1). This algorithm advocated increased investigation and frequency of review for those participants with an abnormal or highly abnormal PlGF result (<100 pg/mL and <12 pg/mL respectively). The final decision regarding further investigation, frequency of further review, and timing of delivery remained with the treating clinician. Participants were eligible for repeat PlGF quantification if four or more weeks had passed from their initial enrollment with a “normal” test, they were still <37 weeks gestation, and they had not been diagnosed with pre-eclampsia but clinical concern persisted. PlGF testing, using any commercially available platform, was not routinely available in any of the hospitals involved either before or during the trial outside of this study.

### Outcomes

The co-primary superiority outcomes were pre-specified composite measures of both maternal and neonatal morbidity (box 1). This co-primary approach was chosen to ensure maternal morbidity was not reduced at the expense of earlier delivery and worse neonatal outcomes. All statistical analyses were based on participants and not events (binary outcomes)—that is, each woman/baby was only counted once in any particular statistical test, as either having the outcome of interest or not. Secondary outcomes included each component of the composite primary outcome reported individually as well as further maternal and neonatal assessments. Maternal and neonatal clinical outcomes were recorded by the local trained research assistant following review of the participants’ and neonates’ clinical healthcare records, 12 weeks after delivery, and final discharge from hospital. All final clinical diagnoses were reviewed by the study monitor at periodic site visits throughout the trial. All pre-eclampsia diagnoses were also reviewed by a central adjudication panel consisting of a clinical doctor and a research midwife, who were masked to site allocation and PlGF result.

Box 1 Components of the morbidity composites*

**Maternal**

- Confirmed placental abruption
- Intensive care admission
- Central nervous system compromise—Generalised tonic clonic seizure due to eclampsia, Glasgow Coma Scale <13, cerebral haemorrhage or infarct, cortical blindness, retinal detachment, transient ischaemic attack, reversible ischaemic neurological deficit
- Cardiorespiratory compromise—Myocardial ischaemia or infarction, blood oxygen saturation <90%, >50% fraction of inspired oxygen (F_I_O_2_) for >1 hour, intubation (other than for caesarean section), pulmonary oedema, need for positive inotrope support
- Haematological compromise—Transfusion of any blood product, platelet count <100×10^9^/L
- Liver compromise—Hepatic dysfunction (ALT or AST >70 IU/L), haematoma, rupture
- Kidney compromise—Acute renal insufficiency (creatinine >150 μmol/L), hemodialysis
- Severe hypertension (systolic blood pressure ≥160 mm Hg on at least one occasion in either antenatal or postnatal period)

**Neonatal**

- Perinatal death or death before hospital discharge
- Neonatal intensive care unit admission for ≥48 hours
- Birthweight ≤5th customised centile*
- Apgar score <7 at 5 minutes
- Umbilical artery acidosis at birth (cord pH <7.2)
- Admission to neonatal unit
- Respiratory distress syndrome
- Intraventricular haemorrhage
- Retinopathy of prematurity
- Confirmed infection (confirmed on blood or cerebrospinal fluid cultures)
- Necrotising enterocolitis

*There is no double counting of events for the co-primary composite outcomes; individuals, not events, are included

As this study was not Health Products Regulatory Authority (HPRA) regulated and did not involve an investigational device, study-specific safety reporting was not mandated by the trial sponsor (University College Cork). There were no study procedures that could have related causality to a serious adverse event (SAE); however, an independent data monitoring committee (DMC) was appointed to protect study participants. Any SAEs, such as perinatal death or profound maternal morbidity, in the intervention phase of the study, were reported immediately to the DMC. No significant clinical concerns with morbidities occurred. As well as monitoring participant safety, the DMC also received regular updates on the progress of the trial every quarter for the purpose of ensuring the quality of data collection, ensuring that the intervention was rolled out according to the randomisation plan and monitoring balance between arms to monitor for potential selection biases and ensured that PlGF testing was not overwhelmingly better or worse than no PlGF testing for maternal morbidity and neonatal morbidity. 

Upon initial data review and before data analysis, it was identified that 60% of neonates were missing the umbilical cord pH variable, which is one of the components of the composite neonatal outcome. Therefore, we elected to modify the neonatal composite to exclude this variable but also to report the neonatal composite as planned initially but restricted to those neonates that had the pH test performed. This decision was supported by the trial DMC and documented in an amendment to the trial protocol before the data were closed for analysis.

### Post hoc additions

In order to facilitate easy comparison of our trial results with those of the UK PARROT trial,^14^ we decided post hoc to include three additional outcomes: time from enrollment until pre-eclampsia diagnosis as well as maternal and neonatal composite outcomes. Together, these outcomes comprised the primary and secondary endpoints of the UK PARROT study. Time to clinician-documented diagnosis was determined by calculating the difference (in days) from the participants’ gestational age at enrollment and randomisation in the trial until their gestational age at the time of diagnosis of pre-eclampsia. Additional post hoc analysis of adverse events and subsequent participant attendance at hospital stratified by PlGF testing were also included.

### Sample size

Sample size calculations were performed approximating binary outcomes by a linear mixed model, and assuming categorical effects for time; random cluster and random cluster by period effects.^21^ The trial statisticians calculated that, with a sample size of 4000 participants and using a two-sided type I error rate of 0.025 (to allow for two co-primary outcomes), the trial would have 80% power to detect a 7% reduction in maternal morbidity (relative risk reduction of 20%) from 35% to 28% in the intervention group. This calculation was based on a reported rate of adverse maternal outcome in the region of 35% in the PELICAN trial.^22^ We assumed an intra-cluster correlation (ICC) in the region of 0.01; but also considered sensitivity to a range of ICC values between 0.005 and 0.05. To allow for the longitudinal nature of the trial, where correlations may differ between observations in the same cluster-period and those measured in different cluster periods, we incorporated cluster-auto correlations (CAC). There was little information to support potential values for the CAC, so we were guided by values in the literature and explored sensitivity across a range of values (0.64, 0.80, and 0.96).^23^ ^24^

For the second co-primary endpoint of adverse neonatal outcomes, based on rates of adverse events in the region of 10%, the trial had 90% power to detect an absolute change in neonatal adverse outcomes of 6%. Due to scarcity of information on the within-cluster correlations, the same values as for the maternal outcome was assumed. Under these assumptions, we constructed power curves, which reveal that, under most anticipated scenarios, the trial will have in the region of 80% power.^24^ ^25^ The power was estimated using an online RShiny App.^26^ ^27^

### Statistical analysis

All participants who completed the trial, aside from those enrolled in the transition periods, were included in the analysis by intention to treat. The statistical analysis of the co-primary endpoints, represented as binary variables, was done using mixed-effects Poisson regression with robust variance estimation to estimate the risk ratios (RR), 95% confidence intervals (CI), and P values (to correct for the misspecification of using the Poisson distribution to model binary outcomes).^28^ This model was used as an alternative to log-linear binomial model because often it does not converge, a known problem. Each model included the intervention variable and time period (8 time periods) as fixed-effects variables and hospital as a random-effects variable. For the co-primary endpoints only, we additionally fitted mixed-effects binomial regression with identify link, including the same variables as the Poisson models, to estimate the risk difference (RD), 95% CI, and P values. To allow for the co-primary endpoints, the results of the co-primary endpoints will be considered statistically significant if the P<0.025 (See appendix on bmj.com for more detail: Additional statistical analysis). All other binary secondary endpoints were analysed using the same approach (appendix: Additional statistical analysis). To estimate the fit of the Poisson and binomial models, we used the default options in Stata; maximum likelihood-based techniques using mean-variance adaptive Gauss-Hermite quadrature. To analyse categorical outcomes mode of delivery (spontaneous delivery, instrumental delivery, elective caesarean section (CS), and emergency CS) and preterm birth (term, preterm, very preterm, and extremely preterm), we used mixed-effects Poisson models choosing one category as a reference. For continuous outcomes gestational age at diagnosis of pre-eclampsia and gestational age at delivery, mixed-effects linear regression was used (with the same adjustments as for the other outcome types).

For the analysis of the time to pre-eclampsia post hoc outcome, we first log-transformed the time from recruitment to pre-eclampsia diagnosis, performed the mixed-effects linear regression using the log-transformed time variable, and report geometric mean ratios. To assess sensitivity to any residual confounding, we repeated the co-primary endpoints models described above and adjusted for the following pre-specified variables: maternal age, body mass index, smoking, ethnic origin, gestational age at booking, type of antenatal care (public/private), and maternal co-morbidities such as chronic hypertension/renal disease, systemic lupus erythematosus, antiphospholipid syndrome, and pre-existing diabetes. To assess sensitivity to assumptions made about nature of time effects and correlations, we conducted an extensive series of sensitivity analyses (appendix: Additional statistical analysis). We calculated the ICCs for all primary and secondary endpoints. Stata 13.1 was used for the statistical analysis apart from the sensitivity analyses for which we used SAS.

### Patient and public involvement

Neither patients nor the public were directly involved in the development of this project, mainly due to time limitations before commencement of the trial. We recognise this as a limitation. Support sites such as Action on Pre-eclampsia and The Pre-eclampsia Foundation were reviewed during the development of the trial protocol, and each of the research ethics committees that reviewed the trial protocol included lay person representation with their unique focus from a PPI perspective. Lay person public representatives were also included as members of the trial steering committee once recruitment had commenced. Embedded within our trial we conducted a prospective nested qualitative cross-sectional study to examine the participants’ experience.^29^

## Results

**Fig 1:**
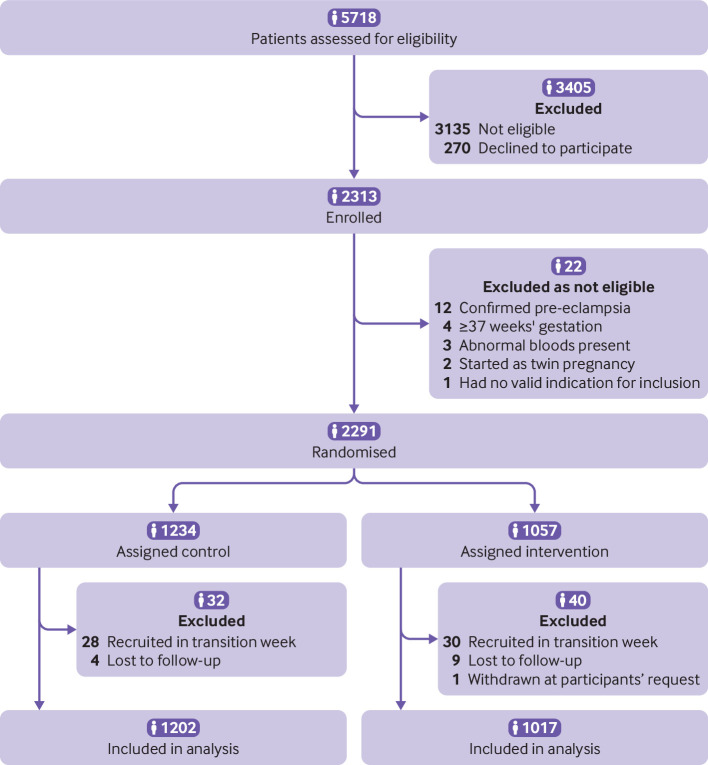
Consort flowchart of trial participants

The PARROT Ireland trial ran for two months, commencing on 29 June 2017, date of first recruited participant, and ceasing on 26 April 2019, date of the final recruited participant. Final participant follow-up concluded in December 2019. During this time, 5718 pregnant women were assessed for eligibility, and 2313 were enrolled in the study (fig 1). Of these, 22 participants (<1%) were subsequently identified as not eligible and excluded before randomisation. Of the 2291 participants randomised, 1234 (54%) were assigned to control and 1057 (46%) to intervention. As per the trial protocol, all those recruited during a transition week were excluded from the analysis, 28 participants (2%) during control transition week and 30 participants (3%) during intervention transition week. A further four participants (<1%) from the control group and nine (<1%) from the intervention group were lost to follow-up. A single participant, assigned to the intervention, requested to be withdrawn from the study. The final numbers for analysis included 1202 participants randomised to control and 1017 randomised to intervention (fig 1). Outcome data were collected until the last recruited participant was 12 weeks after birth of her baby, the last neonate was discharged from hospital, and final clinical outcomes were available. After site close-out visits and data cleaning, the trial dataset was locked in April 2020. The trial statisticians analysed between June and August 2020.

### Participant characteristics

Table 1 provides details of the participants’ baseline characteristics at the first antenatal visit, and table 2 shows their characteristics at the time of enrolment to the study. All participants recruited to the trial were eligible based on the presence of signs or symptoms concerning for evolving pre-eclampsia or placental dysfunction (supplementary tables S1 and S2). The final clinical diagnosis, highlighting an incidence of pre-eclampsia of approximately 14%, as well as any additional adverse diagnosis, are presented for all participants (supplementary table S3).

**Table 1:** Baseline characteristics of participants at time of antenatal booking visit. Values are numbers (percentages) of participants unless stated otherwise

Characteristic	Control (n=1202)	Intervention (n=1017)
Mean (SD) age (years)	31.89 (5.82)	31.83 (5.88)
Ethnicity:		
European	1093 (90.93)	957 (94.10)
African Caribbean	3 (0.25)	0 (0.00)
African	32 (2.66)	16 (1.57)
Bangladeshi	2 (0.17)	2 (0.20)
Indian	16 (1.33)	15 (1.47)
Middle Eastern	9 (0.75)	4 (0.39)
Pakistani	7 (0.58)	6 (0.59)
South East Asian	15 (1.25)	6 (0.59)
Other	25 (2.08)	11 (1.08)
Parity:		
Nulliparous	487 (40.52)	373 (36.68)
Multiparous:	715 (59.48)	644 (63.32)
Previous pre-eclampsia	129 (18.04)	122 (18.94)
Previous stillbirth	11 (1.54)	7 (1.09)
Previous pregnancy loss:		
No	841 (69.97)	690 (67.85)
Yes	361 (30.03)	327 (32.15)
Medical comorbidities:		
Chronic renal disease	32 (2.66)	19 (1.87)
Chronic hypertension	101 (8.40)	65 (6.39)
SLE/APS	5 (0.42)	2 (0.20)
Pre pregnancy diabetes	29 (2.41)	19 (1.87)
Obstetric care:		
Public	1097 (91.26)	932 (91.64)
Private	104 (8.65)	84 (8.26)
Unknown	1 (0.08)	1 (0.10)
Mean (SD) gestation at booking (weeks)	13.17 (3.64)	12.89 (3.49)
Mean (SD) body mass index (kg/m^2^)*	28.3 (6.89)	28.29 (6.76)
Smoking status†:		
Current smoking	143 (11.90)	123 (12.09)
Quit smoking	73 (6.07)	122 (12.00)
Never smoked	982 (81.70)	769 (75.61)
Mean (SD) blood pressure (mm Hg):		
Systolic	120.69 (14.54)	120.62 (12.76)
Diastolic	73.67 (10.16)	74.05 (10.13)
Proteinuria‡:		
No	1071 (89.25)	904 (88.89)
Not done	73 (6.08)	78 (7.67)
Yes:	56 (4.67)	35 (3.44)
Trace	24 (42.86)	17 (48.57)
+1	16 (28.57)	14 (40.00)
+2 or more	16 (28.57)	4 (11.43)

SD=standard deviation. SLE/APS=systemic lupus erythematosus/antiphospholipid syndrome.

Missing data for the following variables (we report the number of women with data on each variable in the two arms). *BMI: 1195 and 1014 women in control and treatment arms respectively. †Smoking status: 1198 and 1014 respectively. ‡Proteinuria: 1200 and 1017 respecitvely.

**Table 2:** Participants’ characteristics at time of enrollment to the study. Values are numbers (percentages) of participants unless stated otherwise

Characteristic	Control (n=1202)	Intervention (n=1017)
Median (IQR) gestation at enrollment (weeks)	33 (29-35)	33 (30-35)
Mean (SD) gestation at enrollment (weeks)	31.8 (4.02)	31.8 (3.98)
Aspirin use in current pregnancy	229 (19.05)	283 (27.83)
Gestational diabetes in current pregnancy	142 (11.81)	109 (10.72)
Location at enrolment:		
Antenatal clinic	278 (23.13)	294 (28.91)
Antenatal ward	251 (20.88)	242 (23.80)
Day ward	417 (34.69)	362 (35.59)
Emergency department	149 (12.40)	99 (9.73)
Labour ward	1 (0.1)	1 (0.1)
Other	106 (8.82)	19 (1.87)
Highest mean (SD) blood pressure recorded in 48 hours before study entry (mm HG)*:		
Systolic	136.29 (18.38)	133.29 (17.49)
Diastolic	83.36 (13.05)	82.47 (13.03)
Highest dipstick level of proteinuria recorded in 48 hours before study entry:		
Trace	151 (12.60)	116 (11.42)
+1	126 (10.52)	100 (9.84)
+2 or more	88 (7.35)	55 (5.41)
None	773 (64.52)	654 (64.37)
Not done	60 (5.01)	91 (8.96)
Fetal scan within two weeks before enrolment:		
No	512 (42.60)	350 (34.41)
Yes	690 (57.40)	667 (65.59)
Suspected fetal growth restriction before enrolment:		
No	330 (47.83)	286 (42.88)
Yes:	360 (52.17)	381 (57.12)
Abdominal circumference <10th centile	233 (64.72)	278 (72.97)
Estimated fetal weight <10th centile	321 (89.17)	335 (87.93)
Umbilical artery PI >95th centile	60 (16.67)	54 (14.17)
AREDF	19 (5.29)	16 (4.20)

IQR=interquartile range. SD=standard deviation. PI=pulsatility index. AREDF=Absence or reversal of end diastolic flow.

* Data for only 1178 and 1009 women in the control and treatment arms respectively.

### Primary outcomes

The co-primary superiority outcomes were composite measures of maternal and neonatal morbidity. The results demonstrate no significant difference in maternal morbidity with the intervention of PlGF testing; 457 (38%) of the 1202 women in the control group versus 330 (32%) of the 1017 women in the intervention group (time and cluster adjusted risk ratio 1.01 (95% CI 0.76 to 1.36) (table 3); adjusted risk difference 0.02 (−0.05 to 0.08)). The overall use of aspirin among enrolled participants in each of our seven hospitals varied, from as low as 6% in one unit to as high as 48% in another. To ensure the lack of difference in primary outcome observed was unrelated to the higher use of aspirin in the intervention group, we conducted post hoc analysis (not shown) adjusting the maternal morbidity composite for use of aspirin and found it to be unaffected.

**Table 3:** Co-primary outcome endpoints of the PARROT Ireland trial: pre-specified composite measures of maternal and neonatal morbidity. Values are numbers (percentages) of participants unless stated otherwise

Endpoint	Control (n=1202)	Intervention (n=1017)	Risk ratio (95% CI)	
			Adjusted Poisson*	Fully adjusted Poisson†
**Maternal morbidity composite**				
No	745 (61.98)	687 (67.55)	1.00 (reference)	1.00 (reference)
Yes	457 (38.02)	330 (32.45)	1.01 (0.76 to 1.36), P=0.92	1.02 (0.80 to 1.31), P=0.87
**Neonatal morbidity composite**				
Based on protocol amendment‡:	n=1202	n=1017		
No	675 (56.16)	535 (52.41)	1.00 (reference)	1.00 (reference)
Yes	527 (43.84)	482 (47.59)	1.03 (0.89 to 1.21), P=0.67	1.03 (0.86 to 1.23), P=0.72
Based on original protocol§:	n=594	n=366		
No	231 (38.9)	112 (30.3)	1.00 (reference)	1.00 (reference)
Yes	363 (61.11)	254 (69.67)	1.06 (0.92 to 1.22), P=0.40	1.04 (0.90 to 1.282), P=0.52

CI=confidence interval.

* Poisson regression models adjusted for time and hospital.

† Poisson regression models adjusted as pre-specified for age, body mass index, smoking, ethnic origin, gestational age at booking, public versus private obstetric care, and maternal comorbities (chronic hypertension, chronic renal disease, systemic lupus erythematosus/antiphospholipid syndrome, and pre-existing diabetes).

‡ 10 of the original 11 variables (see box 1), excluding umbilical artery acidosis at birth.

§ All 11 variables (see box 1), but low numbers due to missing test for umbilical artery acidosis (n=960).

Concurrently, there was no significant difference in neonatal morbidity demonstrated with PlGF testing, with 527 (43%) of the 1202 neonates in the control group versus 484 (47%) of the 1017 neonates in the intervention group (adjusted risk ratio (RR) 1.03 (95% CI 0.89 to 1.21) (table 3), adjusted risk difference (RD) 0.012 (95% CI −0.06 to 0.73)). The neonatal morbidity outcome was unchanged when the full 11 variable composite was examined: 363 (61%) of 594 neonates in the control group versus 254 (69%) of 366 neonates in the intervention group (adjusted RR 1.06 (0.92 to 1.22), P=0.40) (table 3). A series of sensitivity analyses conducted for the primary outcomes showed broadly similar results (supplementary table S4).^30^ ^31^ ^32^

There was one maternal death in the intervention group. Recruited at 36 weeks due to worsening hypertension, the participant had a normal PlGF result and an uncomplicated course for the remainder of her pregnancy. She died 10 weeks after delivery due to acute complications of a known underlying cardiac condition. There were 25 perinatal deaths in total, with more than twice the number of perinatal deaths in the control group (n=17) than in the intervention group (n=8) (RR 0.75 (0.35 to 1.62)) (tables 4 and 5).

**Table 4:** Secondary maternal outcomes. Values are numbers (percentages) of participants unless stated otherwise

	Control (n=1202)	Intervention (n=1017)	Adjusted risk ratio* or mean difference† (95% CI), P value
Final diagnosis of hypertensive disorder of pregnancy	676 (56.24)	507 (49.85)	0.95 (0.91 to 1.00), P=0.05
Median (IQR) gestation at diagnosis of pre-eclampsia (days)	248 (231-260), n=177	252 (235-259), n=138	
Mean (SD) gestation at diagnosis of pre-eclampsia (days)	245.0 (20.51)	246.52 (21.05)	5.01 (−2.55 to 12.57), P=0.19
Use of 1 or more antihypertensive drugs	544 (45.26)	399 (39.23)	0.97 (0.90 to 1.04), P=0.39
Severe hypertension‡ (systolic BP ≥160 mm Hg on ≥1 occasion in antenatal or postnatal period)	401 (33.36)	282 (27.73)	1.04 (0.79 to 1.38), P=0.75
Maternal morbidity by fullPIERS model§:	131 (10.90)	106 (10.42)	1.10 (0.79 to 1.52); P=0.58
Confirmed placental abruption‡	12 (1.00)	6 (0.59)	1.87 (0.21 to 16.28), P=0.57
Intensive care admission‡	1 (0.08)	0 (0.00)	—¶
Central nervous system compromise‡	2 (0.17)	1 (0.10)	—¶
Cardiorespiratory or haematological compromise‡	46 (3.83)	46 (4.52)	1.13 (0.58 to 2.19),P=0.73
Liver or kidney compromise‡	82 (6.82)	65 (6.39)	1.08 (0.62 to 1.88), P=0.79
Progression to severe pre-eclampsia as defined by ACOG	101/177 (57.06)	70/137 (51.09)	0.99 (0.88 to 1.10), P=0.84
Induction of labour**:			
Spontaneous	260 (31.78)	229 (33.58)	1.00 (reference)
Induced	558 (68.22)	453 (66.42)	0.95 (0.84 to 1.07), P=0.38
Model of delivery:			
Spontaneous	454 (37.77)	429 (42.18)	1.00 (reference)
Assisted spontaneous	165 (13.73)	104 (10.23)	0.68 (0.42 to 1.07), P=0.1
Elective caesarean section	273 (22.71)	246 (24.19)	0.90 (0.76 to 1.06), P=0.21
Emergency caesarean section	310 (25.79)	238 (23.40)	0.96 (0.77 to 1.20), P=0.71

CI=confidence interval. IQR=interquartile range. SD=standard deviation. ACOG=American College of Obstetricians and Gynecologists.

* Poisson models adjusted for time and hospital.

† Linear regression model adjusted for time and hospital.

‡ Component of the primary maternal morbidity composite

§ Consists of placental abruption, maternal death, central nervous system compromise, cardiorespiratory compromise, haematological compromise, liver compromise, and kidney compromise. Includes patients with a platelet count <100×10^9^ platelets/L.

¶ Model not run when <5 events.

** Pre-labour caesarean section excluded from this analysis.

**Table 5:** Secondary neonatal outcomes. Values are numbers (percentages) of participants unless stated otherwise

	Control (n=1202)	Intervention (n=1017)	Risk ratio* or mean difference† (95% CI), P value
Fetal growth restriction identified on antenatal ultrasound (<10th centile)	419 (34.86)	405 (39.82)	1.01 (0.97 to 1.06), P=0.56
Median (IQR) gestation at delivery (days)	269 (259-276)	267 (259-275)	
Mean (SD) gestation at delivery (days)	265.14 (18.03)	264.90 (17.30)	−0.83 (−3.93 to 2.27), P=0.60
Perinatal death or death before hospital discharge‡	17 (1.41)	8 (0.79)	0.75 (0.35 to 1.62), P=0.47
Admission to NICU‡	378 (31.45)	314 (30.88)	0.95 (0.75 to 1.20), P=0.65
NICU admission for ≥48 hours‡	291 (24.21)	217 (21.34)	0.81 (0.60 to 1.10), P=0.19
Birth weight ≤5th customised centile‡§	338 (28.12)	307 (30.19)	1.06 (0.96 to 1.16), P=0.26
Apgar score <7 at 5 minutes‡	46 (3.83)	46 (4.52)	1.74 (1.20 to 2.51), P≤0.001
Umbilical artery acidosis at birth‡	115/594 (19.36)	100/366 (27.32)	1.26 (0.87 to 1.84), P=0.22
Respiratory distress syndrome‡	80 (6.66)	76 (7.47)	1.49 (0.65 to 3.42), P=0.35
Intarventricular haemorrhage‡¶	3 (0.25)	6 (0.59)	—
Retinopathy of prematurity‡¶	9 (0.75)	13 (1.28)	—
Confirmed infection‡¶	11 (0.92)	11 (1.08)	—
Necrotising enterocolitis‡¶	2 (0.17)	4 (0.39)	—
Preterm delivery**:			
Term (≥37 weeks)	920 (76.54)	797 (78.37)	1.00 (reference)
Preterm (32-36 weeks)	230 (19.13)	186 (18.29)	0.92 (0.60 to 1.42), P=0.71
Very preterm (28-31 weeks)	44 (3.66)	24 (2.36)	0.88 (0.25 to 3.10), P=0.84
Extremely preterm (<28 weeks)	8 (0.67)	10 (0.98)	3.03 (0.73 to 12.48), P=0.13

CI=confidence interval. IQR=interquartile range. SD=standard deviation. NICU=neonatal intensive care unit.

* Poisson regression adjusted for time and hospital.

† Linear regression adjusted for time and hospital.

‡ Component of primary neonatal morbidity composite.

§ Fetal growth restriction calculated based on actual birth weight, gestation at birth, fetal gender, and maternal ethnicity, parity, and body mass index using the Gestation Related Optimal Weight (GROW) centile calculator (https://www.gestation.net/cc/about.htm).

¶ The mixed-effects Poisson models for these outcomes had convergence issues, possibly due to a small number of events. We tried adjusting for clustering only, tried logistic mixed-model and tried the Laplace option and still noted convergence issues. Therefore we have not reported the RRs because they were not estimable.

** Sub-categorisation of prematurity was added post hoc.

Additional information relating to adverse events and further attendance stratified by PlGF result at randomisation suggests that the test is an accurate reflection of disease severity, especially at the very abnormal threshold, but it did not seem to alter outcomes through modified management or delivery in this study (supplementary table S5).

### Comparison with PARROT UK, post hoc analyses

Using the same composites to define adverse outcomes as the PARROT UK trial, we observed the risk of maternal morbidity (adjusted RR 1.10 (0.79 to 1.52)) or neonatal morbidity (adjusted RR 1.66 (0.81 to 3.42)) to be higher in the intervention arm compared with the control arm, although the evidence is uncertain with wide confidence intervals noted (supplementary table S6). Additionally, when we calculated the time to diagnose pre-eclampsia in our participants, we noted an apparent increase, from seven days in the control group to eight days in intervention group, but the geometric mean difference (GMD) was not statistically significant and had a wide confidence interval (GMD 0.92 (0.56 to 1.49)) (supplementary table S6).

### Secondary outcomes

Secondary outcomes comprising of the individual components of maternal and neonatal morbidity as well as other pre-defined parameters are presented (tables 4 and 5). There was some evidence that the diagnosis of a hypertensive disorder of pregnancy was reduced with the intervention (adjusted RR 0.95 (0.91 to 1.00)), while we found no significant difference between the two groups in the incidence of severe hypertension (adjusted RR 1.04 (0.79 to 1.38)) or gestational age at diagnosis of pre-eclampsia (5.01 ( −2.55 to 12.57)). Although the incidence of placental abruption was higher in the control group, it was not statistically significant with a wide confidence interval reported (adjusted RR 1.87 (0.21 to 16.28)). Further, there was no evidence of a difference between the two groups in the incidence of induction of labour (adjusted RR 0.95 (0.84 to 1.07)), rates of assisted vaginal delivery (0.68 (0.42 to 1.07)), or emergency caesarean delivery (0.96 (0.77 to 1.20)). The likelihood of having an Apgar score <7 at 5 minutes of life was significantly higher in the intervention than control groups (adjusted RR 1.74 (1.20 to 2.51)). All grades of both retinopathy of prematurity and intraventricular haemorrhage were captured, and the absolute numbers of cases were low and likely too small for meaningful interpretation. There was no evidence that median gestational age at delivery differed between the groups, nor did the incidence of preterm birth (adjusted RR 0.92 (0.60 to 1.42)), very preterm birth (0.88 (0.25 to 3.10)), and extremely preterm birth (3.03 (0.73 to 12.48)). Similar numbers in each group required admission to a neonatal intensive care unit (adjusted RR 0.95 (0.75 to 1.20)). The intraclass correlation coefficients are presented in supplementary tables S7-9.

## Discussion

### Principal findings

We set out to examine if the incorporation of PlGF testing to usual clinical care, in women with suspected preterm pre-eclampsia and a singleton pregnancy, improved maternal outcomes without negatively influencing neonatal outcomes. The result of this national multisite RCT demonstrates no significant change in either maternal or neonatal morbidity with the integration of point of care PlGF based testing.

We had estimated a maternal morbidity rate of 35%, based on previous observational studies, and aimed to reduce it to 28%. The baseline incidence of maternal morbidity in the control group was slightly higher than expected at 38%. Notably, there were higher incidences of multiparous participants (27% *v* 19%) and aspirin users (63% *v* 59%) in the intervention group compared with the control group. Multiparity is known to confer a protective effect against pre-eclampsia, and the benefits of aspirin in relative risk reduction for pre-eclampsia has evolved over the past few years.^20^ ^33^ ^34^ Although we supplied a management algorithm to clinicians to incorporate PlGF based testing into clinical practice, this was a suggested algorithm only and adherence was not mandatory. Fidelity to the algorithm was not assessed. Hence this trial demonstrates a pragmatic evaluation of PlGF based testing effectiveness rather than its efficacy.

### Comparison with other studies

Two other RCTs examining the impact of the integration of PlGF testing have been completed to date, both of which were UK based, published in 2019 and reported benefit with the addition of PlGF testing to routine clinical care in women with suspected preterm pre-eclampsia.^14^ ^35^ The INSPIRE trial was conducted at a single site, included 370 participants and used a sFlt-1/PlGF ratio test. It reported that use of the ratio test significantly improved clinical precision without affecting hospital admission rates.^35^ The UK PARROT trial was conducted across 11 sites and enrolled 1035 participants.^14^ It reported PlGF testing to be beneficial based on a reduction in time to diagnose preterm pre-eclampsia as well as a statistically significant reduction in severe maternal adverse outcomes; from 5% (n=24) of women in the concealed testing group versus 4% (n=22) of women in the revealed testing group.

The INSPIRE trial differed to our trial in terms of trial design, PlGF platform used, and primary outcome employed. More comparable to our study is the UK PARROT trial, which used a similar design and PlGF platform. Although not part of our original protocol, we elected to add a results table to facilitate direct comparison of the clinically relevant endpoints (the maternal and neonatal adverse outcomes) between the two PARROT trials (supplementary table S6). Subtle differences exist between the two PARROT trials, specifically in terms of the definition of pre-eclampsia used and the primary outcome employed, but both trials aimed to evaluate PlGF testing in a population-based setting (supplementary table S10). It is possible that the UK and Irish PARROT trials’ differing results may be due to differences in the populations enrolled and examined. Both trials inclusion criteria were almost identical, but a higher proportion of women with suspected fetal growth restriction were recruited to the PARROT Ireland trial (approximately 55% compared with 16%) and the incidence of pre-eclampsia among the UK trial participants was higher (approximately 35% compared with 14%). The net result of these variations is likely a higher incidence of non-hypertensive, small-for-gestational-age (SGA) fetuses in the PARROT Ireland population compared with that of the UK trial. It has been shown that PlGF is not as sensitive in predicting delivery in women with non-hypertensive SGA fetuses, likely due to differing underlying placental pathology.^36^ Potential exists for future analysis of our cohort and stratification by presence or absence of hypertensive complications at time of enrollment to evaluate these population differences further.

### Strengths and limitations of study

We recognise we did not reach recruitment targets, enrolling just 57% of the anticipated 4000 women. The planned date for trial cessation was based on the fixed expiry date of the Triage PlGF devices and controls. As the trial progressed, it became apparent that recruitment was behind projected estimates. Contributory factors to underrecruitment included research staff turnover locally and variation between sites in terms of referral of participants and local clinical pathways. Frequently in research a trial extension can be considered to negate underrecruitment and increase sample size. However, because of the stepped wedge design we used, equal time periods had to be employed in all clusters, and therefore the trial end date was non-negotiable once the trial had moved beyond the first cluster. Despite the underrecruitment, this remains a substantial number of participants and the largest randomised evaluation of interventional PlGF examined to date.

Missing over 50% of the data points for the umbilical cord pH variable is unfortunate, and we fully admit this failing should have been recognised sooner. In Ireland there is no national guideline to stipulate when neonatal cord blood gas sampling should be performed. Following enrollment in the trial, it was not mandated that the neonates of participants have cord pH sampling at time of delivery. As per the trial’s statistical analysis plan, an interim analysis was conducted when 50% of recruitment had occurred and outcome data were available. Given underrecruitment, the interim analysis was not performed until late into the trial course, at the start of 2019. At this point the lack of cord pH information in a large proportion of neonates was identified. In hindsight, a pilot study would have been beneficial both for estimating realistic recruitment targets and highlighting the potential for high volume of missing data for the cord pH variable. For future stepped wedge trials, we advise the interim analysis be planned for mid-way through the trial chronologically rather than based on recruitment targets, as this would have allowed the pH variable to be identified and efforts made to reconcile before trial completion. Examining clinicians’ views regarding PlGF would also be worth considering in future research.

The stepped wedge design of the trial ensured each hospital had an opportunity to experience the intervention and thus remained engaged with the trial and committed to not adopting any off-protocol PlGF testing until the trial ceased. The cluster randomisation allowed a change in clinical management to occur at a hospital level rather than at an individual patient level, facilitating a pragmatic approach to PlGF use. A second strength of the trial is its timing, with the publication of the UK trial results occurring just at the end of our recruitment in April 2019. This enabled equipoise, regarding the potential merits of PlGF testing, to be maintained for the duration of our trial and thus did not influence the participants’ decision to enrol nor clinicians’ decision to refer their patients.

### Conclusion

PARROT Ireland is the largest multicentre RCT of PlGF based testing for women with suspected pre-eclampsia to date. Conducted accross two healthcare systems, it fails to validate the use of PlGF as an interventional test and does not support the results of previous RCTs. Given the immiment assessment of PlGF testing by NICE, we recommend urgent reappraisal of the evidence and postponement of updating of guidelines on PlGF testing until an individual participant data (IPD) meta-analysis is performed.^13^ ^20^ An IPD meta-analysis of participants from both the UK and Irish PARROT trials is feasible, given both trials employed the same online electronic clinical report form templates and collected the same data points. Given the lack of maternal or neonatal clinical benefit demonstrated in our trial, we cannot endorse the use of PlGF testing as a diagnostic adjunct to clinical care in women with suspected pre-eclampsia at present and recommend further research be conducted.

**What is already known on this topic**

Cohort studies have demonstrated high sensitivity and high negative predictive value of placental growth factor (PlGF) based testing in determining the need for delivery in women with suspected pre-eclampsia
Concerns exist that the integration of PlGF testing into routine clinical care may result in an earlier intervention, lowering gestational age at delivery and increasing neonatal morbidity

**What this study adds**

This national, multisite, randomised clinical trial of 2313 pregnant women is the largest randomised control trial to date examining the potential of PlGF as an adjunct to clinical investigation
It demonstrated no evidence of a difference in either maternal or neonatal morbidity when PlGF based testing was integrated into routine clinical care
These results add to the information available currently on the impact of PlGF as an interventional test and do not support the use of PlGF testing as a diagnostic adjunct in women with suspected pre-eclampsia

## Data Availability

Relevant anonymised patient level data available on reasonable request from the corresponding author.

## Notes

PARROT Ireland trial group collaborators. Trial steering committee—S Kappala, N Murphy, E Tully, M Dowling, J O’Leary, R Keane, A Murphy, B McElroy, E Snapes, D Hayes-Ryan, A S Khashan, K Hemming, M Daly, E Ní Bhraonáin, D Devane, D Murphy, A Hunter, A Cotter, F M McAuliffe, J J Morrison, F M Breathnach, E Dempsey, L C Kenny, and K O’Donoghue. Research assistants—N Barry, N Buckley, K Corry, C Dunney, J Courtney, P Gargan, L Heaphy, A Kearney, L Murphy, M Murphy, C Mulcahy, C Ni Laighin, C Nolan, and A Warde.

Contributors: All authors contributed to the overall study design and specific methodologies. LK, DD, and KOD conceived and designed the study with input from all authors. DHR drafted the trial protocol with input from all authors and the trial steering committee. DHR, NM, SK, and KOD were responsible for the day to day conduct of the trial. Data collection at each site was conducted by DJM, AH, JJM, AC, AM, FB, CN, NB, CNL, NB, CD, AK, AW, CM, JC, LH, LM, PG, KC. NM conducted data monitoring and cleaning with assistance from DHR and SK. ASK conducted the statistical analysis with assistance from KH. CE conducted the sensitivity analysis and performed a duplicate analysis of the main outcomes. DHR drafted the manuscript with assistance from ASK, KH, and KOD. All authors have critically read, contributed with inputs and revisions, and approved the final manuscript. The corresponding author attests that all listed authors meet authorship criteria and that no others meeting the criteria have been omitted.

The lead author (the manuscript’s guarantor) affirms that this manuscript is an honest, accurate, and transparent account of the study being reported; that no important aspects of the study have been omitted; and that any discrepancies from the study as originally planned have been explained.

Dissemination to participants and related patient and public communities: The results from the PARROT Ireland trial will be presented both nationally and internationally at conference and published in an established peer reviewed journal. Links to the publication will be provided in all applicable trial registers. Dissemination of results to participants will take place via the media, trial website and relevant participant organisations.

Provenance and peer review: Not commissioned; externally peer reviewed.

## Web extra

Extra material supplied by authors

Web appendix: Supplementary material
