# Supplementary material for: Placental growth factor in assessment of women with suspected pre-eclampsia to reduce maternal morbidity: a stepped wedge cluster randomised control trial (PARROT Ireland)
Source: BMJ. 2021 Aug 13;374:n1857. doi: 10.1136/bmj.n1857 (PMC8361324; doi:10.1136/bmj.n1857)
Supplement: Supplementary file 1 — Web appendix: Supplementary material [file hayd063899.ww.pdf]

Appendix: Supplementary Data

Management Algorithm PARROT Ireland

If patient enroled in ACTIVE arm – integrate additional information from PlGF test as suggested below

Normotensive or mild hypertension: BP up to 149/99 mmHg

<12 pg/ml (Highly abnormal)<br><br>Check PET Bloods	Urgent further investigation<br>Fetal US for growth & doppler<br>If normal repeat doppler weekly<br>CTG from 26 weeks<br>Daily review
≥12 and <100 pg/ml (Abnormal)<br><br>Check PET Bloods	Needs further investigation<br>Fetal growth & doppler within 72 hours<br>At least twice weekly review
≥100 pg/ml (Normal)<br><br>Check PET Bloods	Out patient care –weekly review<br>May have repeat PlGF testing at >4weeks<br>Repeat PET bloods only as per clinical care<br>If <32 weeks or very high risk for PET may review twice weekly

Moderate hypertension: BP 150/100–159/109 mmHg

<12 pg/ml (Highly abnormal)<br><br>Check PET Bloods	Urgent further investigation<br>Fetal US for growth & doppler<br>If normal repeat doppler weekly<br>CTG from 26 weeks<br>Daily Review
≥12 and <100 pg/ml (Abnormal)<br><br>Check PET Bloods	Home if no immediate clinical concern<br>Fetal US growth & Dopplers within 72 hours<br>At least twice weekly review
≥100 pg/ml (Normal)<br><br>Check PET Bloods	Home if no immediate clinical concerns<br>Weekly review<br>May have repeat PlGF testing at >4weeks<br>Repeat PET Bloods only as per clinical care<br>If <32 weeks or very high risk for PET may review twice weekly

Severe hypertension: BP ≥ 160/110mmHg

<12 pg/ml (Highly abnormal)<br><br>Check PET Bloods	Admit. Fetal US for growth & doppler<br>CTG from 26 weeks –Daily CTG<br>If normal repeat doppler weekly<br>If BP stable and PCR <30 consider daily out patient review
≥12 and <100 pg/ml (Abnormal)<br><br>Check PET Bloods	Fetal growth & doppler within 72 hours<br>Consider out patient review once BP controlled –at least twice weekly.
≥100 pg/ml (Normal)<br><br>Check PET Bloods	Out patient review once BP controlled and no immediate concerns –twice weekly<br><br>Repeat PET bloods weekly<br>May have repeat PlGF testing at > 4weeks

Treating clinician has final decision on clinical management

Management Algorithm Version 3.0 25th October 2017

Figure S1: Suggested Management Algorithm

Table S1: Inclusion Criteria

Pregnant women between 20+0 and 36+6 weeks of gestation (inclusive) with a;

- Singleton pregnancy
- Aged 18 years or over
- Able to give informed consent
- Presenting with suspected pre-eclampsia: (one or more of the following)
  - Hypertension
  - Dipstick proteinuria
  - Headache
  - Visual disturbances
  - Epigastric or right upper quadrant pain
  - Increasing oedema
  - Suspected fetal growth restriction
  - If the healthcare provider deems that the woman requires further evaluation for possible pre-eclampsia

Table S2: Exclusion Criteria

- Confirmed pre-eclampsia at point of enrolment;
  "sustained hypertension with systolic BP ≥ 140 or diastolic BP ≥ 90 on at least two occasions at least 4hrs apart) with significant quantified proteinuria (>300mg protein on 24hr collection or urine protein creatinine ratio >30mg/mmol) or abnormal pre-eclampsia bloods"
- ≥37 weeks gestation
- Multiple pregnancy
- Abnormal pre-eclampsia bloods (new onset reduced number of platelets or deranged liver function/renal function tests, identified during routine care prior to enrolment and not attributable to anything other than pre-eclampsia).
- Decision regarding imminent delivery already made
- Lethal fetal abnormality present
- Previous participation in PELICAN trial in a prior pregnancy
- Participation in a conflicting trial at the same time as PARROT Ireland
- Plan to use off protocol PlGF testing

Table S3: Clinical outcome diagnosis 12 weeks post-delivery for women in each arm of the study

	Control n (%) N=1202	Intervention n (%) N=1017
**Final Diagnosis**		
Preeclampsia	177(14.73)	138(13.57)
Gestational Hypertension	324(26.96)	232(22.81)
Gestational Hypertension and SGA	44(3.66)	43(4.23)
Gestational Proteinuria	23(1.91)	25(2.46)
Transient Hypertension	163(13.56)	134(13.18)
Superimposed Preeclampsia (background CHT)	8(0.67)	9(0.88)
Superimposed Preeclampsia (background renal disease)	7(0.58)	0(0.00)
Superimposed preeclampsia (background CHT and renal disease)	4(0.33)	2(0.20)
HELLP	2(0.17)	2(0.20)
Chronic Hypertension only	64(5.32)	39(3.83)
Chronic Hypertension and SGA	20(1.66)	14(1.38)
Chronic Hypertension and Renal Disease	6(0.50)	5(0.49)
Chronic Renal Disease	1(0.08)	2(0.20)
Isolated SGA	213(17.72)	215(21.14)
Suspected SGA only	85(7.07)	103(8.47)
None of these	49(4.08)	52(5.11)
Other	12(1.00)	2(0.20)
Gestation at diagnosis (weeks, days), median (IQR)*	33.79 (29.29, 36.29), n=1162	33.86 (29.29, 36.43), n=981
**Additional Diagnosis**		
No additional diagnosis	966(80.37)	730(71.78)
Eclampsia	2(0.17)	0(0.00)
HELLP	6(0.50)	7(0.69)
DIC	0(0.00)	0(0.00)
ELLP	3(0.25)	1(0.10)
Placental Abruption	12(1.00)	6(0.59)
Transient Hypertension	25(2.08)	53(5.21)
IUGR at delivery	152(12.65)	196(19.27)
IUGR/SGA undetected antenatally	21(1.75)	10(0.98)

Cholecystitis	0(0.00)	3(0.29)
Obstetric Cholestasis	15(1.25)	11(1.08)

**only data on 1,162 and 981 women in the control and treatment arms as some diagnosis were postnatal*

Additional Statistical Analysis

A series of sensitivity analysis were conducted for the primary outcomes to determine if results were sensitive to the Hussey and Hughes model [1]. The Hussey and Hughes model assumes an exchangeable correlation structure; fixed and common time effects, a single time averaged treatment effect, and does not make allowance for the small numbers of clusters [2]. This analysis was considered for the primary outcomes only.

To correct for the potential inflation of the type I error rate due to small number of clusters a small sample correction using the Kenward-Roger method was used throughout these sensitivity analyses. Further sensitivity analysis was undertaken firstly to allow more flexible correlation structures: we considered the addition of a cluster by period random effect; and a discrete time decay correlation structure including a random cluster effect with auto-regressive structure (AR(1)). Secondly, we modelled time effects using splines (with 3 knots); and further allowed for random cluster by intervention effects and finally by estimation of the treatment effect by number of periods since first treatment (to understand if there was a relationship between the duration of exposure to the intervention and outcomes). All models were fitted using the generalised linear mixed model using the 'proc glimmix' function in SAS. To estimate the risk differences and relative risks we used the binomial distribution with an identity link; and the binomial distribution with a log link, respectively.

Results were broadly unsensitive to model choice, although confidence intervals widened with most of the alternative models considered. However, as uncertainty in the primary outcome results was already high in the pre-specified analysis, this additional uncertainty or small widening of the confidence interval, had minimal implications on the overall interpretation of the final result. There was one exception to this: when looking at the effect of treatment by time since roll-out an apparent significant and positive effect of treatment was identified in clusters exposed to between 3 and 4 months of the treatment (under an exchangeable correlation structure for the maternal outcome, and under exchangeable and cluster by period random effect for the neonatal outcome). Under more complicated and realistic correlation structures this did not remain significant.

Multiple testing: Our intention was to report 97.5% CIs so as to allow for the multiplicity of the two co-primary outcomes making a conservative allowance of independence between the two co-primary outcomes. However, on reflection it was felt that this correction was too conservative since the purpose of the trial was to demonstrate effects (in so far as a positive effect on the maternal outcome and no impact on the neonatal outcome) on both the designated primary variables. We therefore report 95% CIs throughout.

Gestational age at delivery: For the outcome gestational age at delivery, which showed evidence of asymmetry and for which a log-transformation did not markedly improve the skewness, and although when we plotted the residuals following the mixed-effects linear model and the distributions were similar in the intervention and control arms, we report estimates using a permutation test (fitting a generalized linear mixed model with random cluster and fixed period effect) to estimate the mean difference and 95% confidence interval as outlined by others [3]. The mean difference in gestational age at delivery between the intervention and control arms was estimated to be -0.77 [95% CI: -4.4 to 1.95] and the p-value was 0.5731, which is not markedly different from the mixed-effects linear regression result.

1. *Hussey MA, Hughes JP. Design and analysis of stepped wedge cluster randomized trials. Contemp Clin Trials. 2007;28(2):182-191.*
2. *Li F, Hughes JP, Hemming K, Taljaard M, Melnick ER, Heagerty PJ. Mixed-effects models for the design and analysis of stepped wedge cluster randomized trials: An overview. Statistical Methods in Medical Research.0(0):0962280220932962.*
3. *Thompson J, Davey C, Hayes R, Hargreaves J, Fielding K. Permutation tests for stepped-wedge cluster-randomized trials. The Stata Journal. 2019;19(4):803-819.*

Table S4: Additional Sensitivity Analysis

**PARROT Ireland study - results**	**SAS**		**Small sample correction -** Kenward-Roger			
	**Outcome:**	**Maternal**	prevalence of outcome= 35.47%			
**Sensitivity analysis**						
**Correlation structure**	**Type of treatment effect**	**Treatment effect**	**Lower 95% confidence interval**	**Upper 95% confidence interval**	**p value**	
Block exchangeable (random effects (cluster, period))	RR	1.028	0.8141	1.2982	0.8111	
Block exchangeable (random effects (cluster, period))	RD	0.01345	-0.07022	0.09711	0.7468	
Block exchangeable (random effects (cluster, period))	OR	1.0573	0.7267	1.5381	0.7652	
Discrete time decay (AR(1))	RR	1.0417	0.8074	1.3441	0.7456	
Discrete time decay (AR(1))	RD	0.01483	-0.07611	0.1058	0.7424	
Discrete time decay (AR(1))	OR	1.0768	0.7152	1.6214	0.7151	
**Additional sensitivity analysis - splines (3 knots)**						
**Correlation structure**	**Type of treatment effect**	**Treatment effect**	**Lower 95% confidence interval**	**Upper 95% confidence interval**	**p value**	
Exchangeable (random effects (cluster))	RR	0.9998	0.8309	1.203	0.998	
Exchangeable (random effects (cluster))	RD	0.01319	-0.05265	0.07904	0.6944	
Exchangeable (random effects (cluster))	OR	1.0275	0.7638	1.3822	0.8577	
Block exchangeable (random effects (cluster, period))	RR	1.0409	0.8637	1.2544	0.6737	
Block exchangeable (random effects (cluster, period))	RD	0.01848	-0.05014	0.08711	0.5973	

Block exchangeable (random effects (cluster, period))	OR	1.0713	0.7895	1.4537	0.6582	
Discrete time decay (AR(1))	RR	1.0675	0.856	1.3313	0.5582	
Discrete time decay (AR(1))	RD	0.02907	-0.04895	0.1071	0.4624	
Discrete time decay (AR(1))	OR	1.1233	0.7884	1.6004	0.5164	
**Additional sensitivity analysis - Cluster by intervention effects**						
**Correlation structure**	**Type of treatment effect**	**Treatment effect**	**Lower 95% confidence interval**	**Upper 95% confidence interval**	**p value**	
Exchangeable (random effects (cluster))	RR	1.0443	0.8192	1.3313	0.7116	
Exchangeable (random effects (cluster))	RD	0.01954	-0.06829	0.1074	0.6447	
Exchangeable (random effects (cluster))	OR	1.0846	0.7382	1.5936	0.6632	
Block exchangeable (random effects (cluster, period))	RR	1.0448	0.8076	1.3516	0.7251	
Block exchangeable (random effects (cluster, period))	RD	No convergence				
Block exchangeable (random effects (cluster, period))	OR	1.0785	0.7162	1.624	0.7039	
**Additional sensitivity analysis - Treatment and number of periods since first treatment (Categorised)**	**Type of treatment effect**	**Treatment effect**	**Lower 95% confidence interval**	**Upper 95% confidence interval**	**p value**	
**Correlation structure**	Reference=control group					
Exchangeable (random effects (cluster)) - RR	0 - 2 periods	1.0344	0.8574	1.2479	0.7238	

	3 - 4 periods	0.6774	0.4768	0.9622	0.0296	
	5 - 6 periods	0.8972	0.5857	1.3742	0.6177	
Exchangeable (random effects (cluster)) - RD	0 - 2 periods	No convergence				
	3 - 4 periods	No convergence				
	5 - 6 periods	No convergence				
Exchangeable (random effects (cluster)) - OR	0 - 2 periods	1.0636	0.7792	1.4516	0.6976	
	3 - 4 periods	0.5937	0.3438	1.025	0.0613	
	5 - 6 periods	0.8569	0.4259	1.724	0.6646	
Block exchangeable (random effects (cluster, period)) -RR	0 - 2 periods	1.04	0.8331	1.2982	0.7193	
	3 - 4 periods	0.6919	0.4633	1.0332	0.0707	
	5 - 6 periods	0.9033	0.5426	1.5038	0.6846	
Block exchangeable (random effects (cluster, period)) - RD	0 - 2 periods	No convergence				
	3 - 4 periods	No convergence				
	5 - 6 periods	No convergence				
Block exchangeable (random effects (cluster, period)) - OR	0 - 2 periods	1.0654	0.7373	1.5394	0.7285	
	3 - 4 periods	0.6138	0.3255	1.1575	0.1279	
	5 - 6 periods	0.8498	0.365	1.9782	0.6963	
Discrete time decay (AR(1)) - RR	0 - 2 periods	1.1101	0.8721	1.4129	0.3871	

	3 - 4 periods	0.8255	0.5032	1.3541	0.4363	
	5 - 6 periods	1.1667	0.5954	2.2863	0.6393	
Discrete time decay (AR(1)) -RD	0 - 2 periods	0.02753	-0.06113	0.1162	0.5356	
	3 - 4 periods	-0.03574	-0.1954	0.124	0.6539	
	5 - 6 periods	0.05118	-0.1805	0.2828	0.6562	
Discrete time decay (AR(1)) - OR	0 - 2 periods	1.1644	0.7843	1.7286	0.4421	
	3 - 4 periods	0.7965	0.375	1.6917	0.5444	
	5 - 6 periods	1.2931	0.4495	3.7195	0.6217	
**ICC**				SAS output		
**Correlation structure**				**ClusterID1**	0.008466	
Exchangeable (random effects (cluster))	Between period	0.036702418		**Residual**	0.2222	
Block exchangeable (random effects (cluster, period))	Within period	0.043702824		**ClusterID1**	0.007983	
Block exchangeable (random effects (cluster, period))	Between period	0.034590486		**ClusterID* TimePeriod**	0.002103	
Block exchangeable (random effects (cluster, period))	Cluster autocorrelation	0.791493159		**Residual**	0.2207	
Discrete time decay (AR(1))	Within period	0.041282231		**Variance**	**ClusterID 1**	0.0095
Discrete time decay (AR(1))	Rate	0.990501		**AR(1)**	**ClusterID 1**	0.9153
				**Residual**		0.2206

Table S5: Outcomes for adverse events and further attendance by PlGF Testing

	Control	***Intervention Very Abnormal <12 pg/ml***	***Intervention Abnormal 12-100 pg/ml***	***Intervention Normal >100 pg/ml***
	n (%) N=1202	***n (%) N=108 (10.6)***	***n (%) N=320 (31.5)***	***n (%) N=589 (57.9)***
Adverse Event				
Maternal Death	*0*	*0*	*0*	*1*
Maternal Stroke	*0*	*0*	*0*	*0*
Eclampsia	*2*	*0*	*0*	*0*
Intrauterine Fetal Death	*10*	*3*	*1*	*1*
Neonatal Death	*7*	*1*	*0*	*2*
Maternal primary endpoint	*457 (38.02)*	*64 (59.26)*	*143 (44.69)*	*123 (20.88)*
Neonatal primary endpoint	*527 (43.84)*	*94 (87.04)*	*144 (45.0)*	*244 (41.43)*
Further attendance ** median (IQR)*				
*GP Visits***	*0 (0, 1), n=955*	*0 (0, 0), n=92*	*0 (0, 1), n=265*	*0 (0, 1), n=496*
ANC Visits	*2 (1, 4)*	*0 (0, 1)*	*2 (0.5, 3)*	*3 (2, 5)*
Day Ward Visits	*1 (0, 2)*	*0 (0, 2)*	*1 (0, 3)*	*1 (0, 2)*
Emergency out of hours visits	*0 (0, 1)*	*0 (0, 0)*	*0 (0, 1)*	*0 (0, 1)*
Fetal Ultrasounds	*1 (0, 3)*	*2 (1, 4.5)*	*2 (1, 3)*	*2 (1, 5)*
Total inpatient nights	*5 (3, 9)*	*9.5 (6, 15)*	*6 (4, 10)*	*5 (3, 7)*

**Attendance following enrollment in the trial until final discharge post- delivery*
***There were missing data for the GP visits variable*

Table S6: Comparison of Maternal and Neonatal Adverse Outcomes as per PARROT UK

	**Control UK n (%) (n=446)**	**Intervention UK n (%) (n=573)**	**AOR* (95% CIs), p-Value**	**Control Ireland n (%) (n=1202)**	**Intervention Ireland n (%) (n=1017)**	**Adjusted RR (95% CIs), p-Value**
**Maternal adverse outcomes****	24(5)	22(4)	0·32 (0·11–0·96) p=0·04	131 (10.90)	106 (10.42)	1.10 (0.79, 1.52) p=0.58
**Perinatal adverse outcomes*****	63(14)	86(15)	1·45 (0·73–2·90)	85 (7.07)	87 (8.55)	1.66 (0.81, 3.42) p=0.17
**Number diagnosed with pre-eclampsia**	155 (35)	205 (36)	Geometric mean difference (95% CI), p-value	177 (14.73)	138 (13.57)	Geometric mean difference (95% CI), p-value****
**Median time (days) to diagnose pre-eclampsia (IQR)**	4.1 (0.8-14.7)	1.9 (0.5-9.2)	0·36, (0·15–0·87) p=0·03	7 (1-25)	8 (1-23)	0.92 (0.56, 1.49), p=0.73

**adjusted Odds Ratios are reported as per the results of the PARROT UK trial*

***Maternal deaths, Eclampsia, Stroke, Parenteral infusion of third-line antihypertensive required, Myocardial infarction, Blood oxygen saturation <90%, Intubation required (other than for caesarean section), Pulmonary oedema, Transfusion of blood products required, Platelet count <50 × 10^9 platelets per L, Hepatic dysfunction, Severe acute kidney injury, Dialysis required, Placental abruption*

****Any grade of intraventricular haemorrhage, Seizure, Any grade of retinopathy of prematurity, Respiratory distress syndrome, Bronchopulmonary dysplasia, Necrotising enterocolitis (stage 2 or 3)*

*****Mixed-effects linear regression model, with log-transformed time to diagnosis and adjusted for time and hospital*

Table S7: The intraclass correlation coefficients of the co-primary endpoints

**Endpoint**	**ICC ***	**SE**	**95% CI**
Maternal endpoint	0.0321	0.0186	0.0102, 0.0967
Neonatal endpoint	0.0075	0.0062	0.0015, 0.0374

ICC: Intraclass correlation; SE: standard error.

**The ICCs were estimated following the regression models for the co-primary endpoints. However, this was done following a mixed-effects linear regression instead of mixed-effects Poisson regression.*

Table S8: The intraclass correlation coefficients of the maternal secondary endpoints

**Endpoint**	**ICC ***	**SE**	**95% CI**
Final diagnosis of hypertensive disorder of pregnancy	0.0259	0.0162	0.0075, 0.0854
Gestation at diagnosis of pre-eclampsia in days, mean (SD)	0.0064	0.0144	0.0001, 0.3586
Use of 1 or more antihypertensive drugs	0.0112	0.0080	0.0027, 0.0446
Severe hypertension<br>(systolic BP ≥ 160 mmHg on at least one occasion)	0.0417	0.0232	0.0137, 0.1197
**Maternal morbidity by fullPIERS model**			
Confirmed placental abruption	0.0017	0.0041	0.0000, 0.1568
Cardiorespiratory Compromise/Haematological Compromise	0.0051	0.0043	0.0010, 0.0263
Liver/Kidney Compromise	0.0007	0.0032	0.0000, 0.8226
Progression to severe pre-eclampsia as defined by ACOG	0.0809	0.0581	0.0187, 0.2894
**Induction of labour**			
Spontaneous labour [reference]			
Induced labour	0.0038	0.0044	0.0004, 0.0362
**Model of delivery**			
Spontaneous delivery [reference]			
Assisted spontaneous delivery	0.0104	0.0097	0.0016, 0.0628
Elective Caesarean Section delivery	0.0183	0.0126	0.0047, 0.0686
Emergency Caesarean section delivery	0.0355	0.0215	0.0107, 0.1117

ICC: Intraclass correlation; SE: standard error.

**The ICCs were estimated following the regression models for the maternal secondary endpoints. However, this was done following a mixed-effects linear regression instead of mixed-effects Poisson regression.*

Table S9: The intraclass correlation coefficients of the neonatall secondary endpoints

**Endpoint**	**ICC ***	**SE**	**95% CI**
Fetal growth restriction identified on antenatal ultrasound (<10th centile)	0.0283	0.0168	0.0087, 0.8816
Gestation at delivery, mean (SD)	0.0073	0.0067	0.0012, 0.0434
Perinatal death or death before hospital discharge	0.0024	0.0029	0.0002, 0.0245
Admission to NICU	0.0254	0.0150	0.0079, 0.0790
NICU admission for ≥48 hrs	0.0093	0.0066	0.0023, 0.0372
Birthweight ≤ 5th customised centile	0.0081	0.0063	0.0017, 0.0371
Apgar score <7 at 5 minutes	0.0049	0.0047	0.0007, 0.0317
Umbilical artery acidosis at birth	0.0088	0.0090	0.0012, 0.0631
Respiratory distress syndrome	0.0145	0.0099	0.0038, 0.0540
Interventricular haemorrhage	0.0007	0.0022	0.0000, 0.2320
**Preterm delivery**			
Term birth (37+) [reference]			
Preterm birth (32-36)	0.0032	0.0049	0.0002, 0.0585
Very preterm birth (28-31)	0.0013	0.0031	0.0000, 0.1139
Extremely preterm birth (<28)	0.0003	0.0037	0.0000, 1.0000

ICC: Intraclass correlation; SE: standard error.

**The ICCs were estimated following the regression models for the neonatal secondary endpoints. However, this was done following a mixed-effects linear regression instead of mixed-effects Poisson regression.*

Table S10: Similarities and Differences in PARROT UK versus PARROT Ireland RCTs

	**UK**	**Ireland**
Date of commencement of Trial	June 2016	June 2017
Date of cessation of Trial	October 2017	April 2019
Number of clusters (Maternity Units)	11	7
Duration of trial (months)	17	22
Duration of each block (weeks)	6	12
Gestational age of participants at recruitment	20-36+6 weeks	20-36+6 weeks
Inclusion Similarities	new-onset or worsening of existing hypertension<br>dipstick proteinuria<br>epigastric or right upper-quadrant pain<br>headache with visual disturbances<br>fetal growth restriction	new-onset or worsening of existing hypertension<br>dipstick proteinuria<br>epigastric or right upper-quadrant pain<br>headache with visual disturbances<br>fetal growth restriction
Inclusion differences	If abnormal maternal blood tests that were suggestive of disease (thrombocytopenia or hepatic or renal dysfunction) participants were still eligible for inclusion	If abnormal maternal blood tests that were suggestive of disease, participants were not included
Number of Participants Enrolled	1035	2313
Guidelines used for diagnosis of Preeclampsia	ISSHP 2014	NICE 2010
Primary Outcome	Time to diagnose confirmed preeclampsia	Maternal and Neonatal Morbidity
